# Genomes of Neutrophilic Sulfur-Oxidizing Chemolithoautotrophs Representing 9 Proteobacterial Species From 8 Genera

**DOI:** 10.3389/fmicb.2019.00316

**Published:** 2019-02-25

**Authors:** Tomohiro Watanabe, Hisaya Kojima, Kazuhiro Umezawa, Chiaki Hori, Taichi E. Takasuka, Yukako Kato, Manabu Fukui

**Affiliations:** ^1^Institute of Low Temperature Science, Hokkaido University, Sapporo, Japan; ^2^Max Planck Institute for Terrestrial Microbiology, Marburg, Germany; ^3^Research Faculty of Engineering, Hokkaido University, Sapporo, Japan; ^4^Research Faculty of Agriculture, Hokkaido University, Sapporo, Japan

**Keywords:** sulfur-oxidizing bacteria, ‘*Sulfuricellaceae*’, *Thiobacillaceae*, *Sterolibacteriaceae*, comparative genomics

## Abstract

Even in the current era of metagenomics, the interpretation of nucleotide sequence data is primarily dependent on knowledge obtained from a limited number of microbes isolated in pure culture. Thus, it is of fundamental importance to expand the variety of strains available in pure culture, to make reliable connections between physiological characteristics and genomic information. In this study, two sulfur oxidizers that potentially represent two novel species were isolated and characterized. They were subjected to whole-genome sequencing together with 7 neutrophilic and chemolithoautotrophic sulfur-oxidizing bacteria. The genes for sulfur oxidation in the obtained genomes were identified and compared with those of isolated sulfur oxidizers in the classes *Betaproteobacteria* and *Gammaproteobacteria*. Although the combinations of these genes in the respective genomes are diverse, typical combinations corresponding to three types of core sulfur oxidation pathways were identified. Each pathway involves one of three specific sets of proteins, SoxCD, DsrABEFHCMKJOP, and HdrCBAHypHdrCB. All three core pathways contain the SoxXYZAB proteins, and a cytoplasmic sulfite oxidase encoded by *soeABC* is a conserved component in the core pathways lacking SoxCD. Phylogenetically close organisms share same core sulfur oxidation pathway, but a notable exception was observed in the family ‘*Sulfuricellaceae*’. In this family, some strains have either core pathway involving DsrABEFHCMKJOP or HdrCBAHypHdrCB, while others have both pathways. A proteomics analysis showed that proteins constituting the core pathways were produced at high levels. While hypothesized function of HdrCBAHypHdrCB is similar to that of Dsr system, both sets of proteins were detected with high relative abundances in the proteome of a strain possessing genes for these proteins. In addition to the genes for sulfur oxidation, those for arsenic metabolism were searched for in the sequenced genomes. As a result, two strains belonging to the families *Thiobacillaceae* and *Sterolibacteriaceae* were observed to harbor genes encoding ArxAB, a type of arsenite oxidase that has been identified in a limited number of bacteria. These findings were made with the newly obtained genomes, including those from 6 genera from which no genome sequence of an isolated organism was previously available. These genomes will serve as valuable references to interpret nucleotide sequences.

## Introduction

**FIGURE 1:**
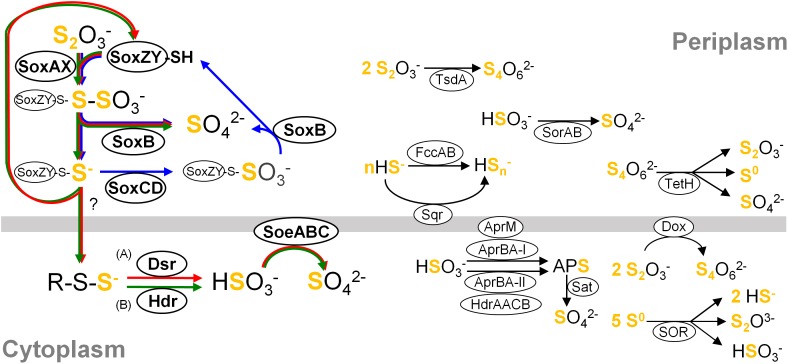
Simplified overview of major components constituting sulfur oxidation pathways in the classes *Betaproteobacteria* and *Gammaproteobacteria*. For clarity, reactions are not shown with exact stoichiometry. Pathways occurring in different organisms are summarized in this single picture, and a variety of pathways could consist of various combinations of these components connected by their reactants and products. Core pathways are highlighted with arrows in blue (cSox), red (Sox-Dsr-Soe) and green (Sox-Hdr-Soe), respectively. (A) The substrate for Dsr system is the persulfurated DsrC protein. (B) The substrate for HdrCBAHypHdrCB was postulated as persulfides (Koch and Dahl, 2018). Abbreviations are as follows: Sqr, sulfide:quinone oxidoreductase; Fcc, flavocytochrome *c* sulfide dehydrogenase; Sox, sulfur-oxidizing enzyme system; DoxDA, thiodulfate:quinone oxidoreductase; TetH, tetrathionate hydrolase; SOR, sulfur oxygenase reductase; Dsr, dissimilatory sulfite reductase; Apr, dissimilatory adenylylsulfate reductase; Hdr, heterodisulfide reductase; Sat, sulfate adenylyltransferase; Soe, sulfite-oxidizing enzyme; Sor, sulfur dehydrogenase. Details of these components are described in references cited in “*Distribution of genes for sulfur oxidation*” in main text.

Phylogenetically diverse bacteria have the capability of utilizing sulfur compounds as electron donors for respiration or phototrophic carbon fixation (Muyzer et al., 2013; Sorokin et al., 2013; Dahl, 2017). These sulfur-oxidizing bacteria have sulfur oxidation pathways consisting of various components (examples are shown in Figure 1), and distribution of the genes for sulfur oxidation has been investigated in diverse prokaryotic genomes (e.g., Meyer and Kuever, 2007; Meyer et al., 2007; Gregersen et al., 2011; Watanabe et al., 2014; Scott et al., 2018). These bacteria are also involved in the carbon and nitrogen cycles, playing crucial roles in natural environments (e.g., Mattes et al., 2013; Prokopenko et al., 2013; Herrmann et al., 2015; Dyksma et al., 2016; Lau et al., 2016). With their physiological functions, these bacteria have been intensively studied for applications such as water treatment, bioleaching and bioremediation (Pokorna and Zabranska, 2015; Lin et al., 2018). Recent advances in DNA sequencing technology have expanded our knowledge of uncultured microorganisms that are presumably oxidizing sulfur compounds (e.g., Kojima et al., 2015a; Mußmann et al., 2017; Tian et al., 2017; Hausmann et al., 2018). However, even in metagenomic studies, the interpretation of the resulting data is fundamentally dependent on knowledge obtained from studies on cultured organisms. In addition, pure culture-based experiments are also indispensable for verifying new concepts proposed by culture-independent studies. Because the majority of microorganisms remain unculturable, it is important to expand the variety of culturable isolates and to take full advantage of the available pure cultures.

*Sulfuricella denitrificans* skB26 and *Sulfuritalea hydrogenivorans* sk43H are sulfur-oxidizing bacteria that were isolated from a same freshwater lake using the same defined medium (Kojima and Fukui, 2010, 2011). The isolation of these strains marked the beginning of a series of studies performed to obtain pure cultures of novel species of neutrophilic sulfur-oxidizing bacteria. In these studies, variations of the same medium were used with minor changes in composition to cultivate diverse sulfur oxidizers, primarily from freshwater environments. The use of this approach led to descriptions of 11 new species, which established 10 genera in the classes *Betaproteobacteria* and *Gammaproteobacteria* (Kojima and Fukui, 2010, 2011, 2014, 2016; Kojima et al., 2015b, 2016, 2017a,b; Watanabe et al., 2015, 2016b,c). The description of these genera resulted in some proposals for reclassification at higher taxonomic levels (Watanabe et al., 2014, 2015; Kojima et al., 2015b, 2017b). Among these sulfur oxidizers, complete genome sequences have been reported for the type strains of four species, *Sulfuricella denitrificans*, *Sulfuritalea hydrogenivorans*, *Sulfurifustis variabilis*, and *Sulfuricaulis limicola* (Watanabe et al., 2014; Umezawa et al., 2016).

In addition to the impact made on taxonomy, the isolation and characterization of these sulfur-oxidizing bacteria have contributed to a better understanding of the structure and function of microbial communities. Their gene sequences have served as reliable references to interpret nucleotide sequences retrieved from environments. As reviewed and demonstrated in previous studies, 16S rRNA gene sequences of the genera *Sulfuricella* and *Sulfuritalea* have been detected in various natural and engineered freshwater environments (Watanabe et al., 2012, 2014, 2016a,c, 2017). The relatives of *Sulfuricella* and *Sulfuritalea* have also been detected based on detection of sequences related to other genes of these genera (Watanabe et al., 2013; Kojima et al., 2014; Herrmann et al., 2015, 2017; Lau et al., 2016; Kumar et al., 2017, 2018; Feng et al., 2018; Luo et al., 2018). This is also the case for the other genome-sequenced species of *Sulfurifustis* and *Sulfuricaulis* (Watanabe et al., 2016a; Herrmann et al., 2017; Kumar et al., 2018; Włodarczyk et al., 2018). Furthermore, detailed inspections of the genomes of *Sulfuricella* and *Sulfuritalea* revealed the presence of characteristic genes for arsenic metabolism, *arxAB* and *arrAB* (Watanabe et al., 2014, 2017). These findings motivated studies regarding the previously unrecognized functions of these organisms. Consequently, arsenate respiration by *Sulfuritalea hydrogenivorans* sk43H was demonstrated (Watanabe et al., 2017), and *arxA* gene sequences closely related to that of *Sulfuricella denitrificans* skB26 were detected from freshwater environments (Ospino et al., 2018). Genome sequences also provide a basis for proteomic analyses. Based on the genome sequence of *Sulfuricella denitrificans* skB26, expression pattern of proteins involved in sulfur oxidation was investigated in this approach (Watanabe et al., 2012).

As shown by the previous studies mentioned above, the genome sequences of isolated and characterized sulfur oxidizers provide a great deal of valuable information for microbiology and related fields. In this study, the genomes of the remaining 7 species were sequenced along with those of two newly isolated sulfur oxidizers. These organisms represent 8 genera in the classes *Betaproteobacteria* and *Gammaproteobacteria*, including 6 genera whose genomes have not been available until now. The obtained sequences were used for comparative genomics with other sulfur oxidizers to obtain insights into the mechanisms of their sulfur metabolism. Furthermore, the expression of some notable genes was confirmed in proteomic experiments.

## Materials and Methods

### Isolation and Characterization of Novel Sulfur-Oxidizing Bacteria

Sulfur-oxidizing bacteria capable of chemolithoautotrophic growth were newly isolated and characterized. One of the novel strains, strain HaS4, was isolated from the water of Lake Harutori (Kubo et al., 2014). The other strain, strain J5B, was isolated from Jozankei hot spring via an enrichment culture established in a previous study (Kojima et al., 2017a). Prior to the detailed characterization of these microbes, the phylogenetic positions of the isolates were identified by sequencing their PCR-amplified 16S rRNA genes. The physiological characterizations were made in consideration of the features of their close relatives that were identified by the phylogenetic analysis. More detailed procedures for the isolation and characterization of these strains are described in the Supplementary Material.

### Genome Sequencing

The newly isolated strains were subjected to whole-genome sequencing, along with other strains that have been isolated and maintained in our laboratory (Table 1). Genomic DNA from each strain was extracted with a Wizard Genomic DNA Purification Kit (Promega). For sequencing, different methods were used depending on the strains, as described below and summarized in Table 2.

**Table 1:** General features of strains subjected to whole genome sequencing in this study.

		Temperature range for	pH range	Hetrotrophic	Anaerobic
Strain	Family	growth (°C)	for growth	growth	growth	References
*Sulfurisoma sediminicola* BSN1	*Sterolibacteriaceae*	8–34	6.8–8.8	+	+	Kojima and Fukui, 2014
*Sterolibacteriaceae* sp. J5B	*Sterolibacteriaceae*	28–50	5.8–8.7	+	+	This study
*Sulfuriferula multivorans* TTN	‘*Sulfuricellaceae*’^∗^	8–32	5.3–8.6	+	+	Watanabe et al., 2015
*Sulfuriferula thiophila* mst6	‘*Sulfuricellaceae*’^∗^	5–34	5.8–8.1	-	-	Watanabe et al., 2016b
*Sulfurirhabdus autotrophica* BiS0	‘*Sulfuricellaceae*’^∗^	0–32	5.2–8.1	-	-	Watanabe et al., 2016c
*Sulfuritortus calidifontis* J1A	*Thiobacillaceae*	15–48	6.2–8.7	-	+	Kojima et al., 2017a
*Sulfurivermis fontis* JG42	*Thioalkalispiraceae*	25–50	6.1–8.9	-	+	Kojima et al., 2017b
*Sulfuriflexus mobilis* ask1	*Granulosicoccaceae*	5–34	6.4–8.7	-	+	Kojima and Fukui, 2016
*Thiomicrorhabdus* sp. HaS4	*Piscirickettsiaceae*	0–25	6.2–8.8	-	-	This study


*^∗^According to GTDB taxonomy (see text for detail).*

**Table 2:** General features of genome sequences obtained in this study (deposited under BioProject PRJDB7001).

Strain	Sequencing platform	Total length (bp)	No. of contig	Topology of contigs	DDBJ/Genbank accession number
BSN1	Roche FLX + Sanger	2,995,111	8	Linear	BHVV01000000
TTN	Illumina HiSeq	3,616,383^∗^	92	Linear	BGOW01000000
mst6	Illumina HiSeq	2,834,181^∗^	55	Linear	BHGL01000000
BiS0	Illumina HiSeq	3,878,683^∗^	174	Linear	BHVT01000000
J1A	PacBio RS II	2,720,636	1	Circular	AP018721
JG42	PacBio RS II	3,246,214	1	Circular	AP018724
aks1	PacBio RS II	3,111,340	1	Circular	AP018725
HaS4	PacBio RS II	2,537,035	2	Circular	AP018722, AP018723
J5B	PacBio RS II	2,811,460	3	Circular	AP018718-AP018720


*^∗^Including estimated length of assembly gaps within scaffolds.*

The genome of *Sulfurisoma sediminicola* BSN1 was sequenced with the Genome Sequencer FLX System (Roche). The library preparation, FLX sequencing and subsequent PCR-based gap closing were performed as described previously (Watanabe et al., 2012). The genomes of three strains of the genera *Sulfuriferula* and *Sulfurirhabdus* were sequences using an Illumina HiSeq platform. These genera were formerly classified as members of the family ‘*Sulfuricellaceae*’ (Watanabe et al., 2014, 2015), which has subsequently been integrated with the family *Gallionellaceae* (Boden et al., 2017a). However, in this study, the term ‘*Sulfuricellaceae*’ is used to refer to the lineage consisting of these genera and *Sulfuricella*. The presence of the family ‘*Sulfuricellaceae*’ independent from *Gallionellaceae* is shown in the GTDB taxonomy^1^, which is based on extensive phylogenetic analysis using whole genomes (Parks et al., 2018). The genome sequences of *Sulfuriferula* and *Sulfurirhabdus* were obtained by paired-end sequencing, and the outputs of the Velvet assembler were directly used for further analyses. The genome sequences of the other strains were obtained using the PacBio RS II system with essentially the same methods as described previously (Umezawa et al., 2016). From the resulting linear contigs, circular contigs were manually constructed by connecting both ends of each linear contig based on duplicated sequences that appeared at the terminal regions.

### Comparative Genome Analysis and the Identification of Genes for Sulfur Oxidation

For the comparative genome analysis, the genome sequences of sulfur oxidizers belonging to the classes *Betaproteobacteria* and *Gammaproteobacteria* were obtained from National Center for Biotechnology Information (as of June 2017). The organisms were selected for their ability for growth via the oxidation of inorganic sulfur compounds, as demonstrated by experiments with pure cultures. For the newly sequenced genomes, protein-coding sequences were identified using the RAST server (Aziz et al., 2008). In the newly and previously sequenced genomes, the genes encoding for proteins involved in sulfur oxidation (Sqr, FccAB, SoxXYZABCD, DoxDA, TsdA, TetH, Sor, DsrABEFHCMKJOP, AprBA, AprM, HdrAACB, Sat, HdrCBAHypHdrCB, SreABC, SoeABC, and SorAB) were identified based on sequence similarity, with the proteins listed in Supplementary Table S1 used as queries.

### Proteomic Analysis

A proteomic analysis was performed for the strains *Sulfuriferula thiophila* mst6, *Sulfurirhabdus autotrophica* BiS0, and *Sulfurifustis variabilis* skN76. These strains were cultured in a bicarbonate-buffered medium that contained thiosulfate as the sole electron donor for chemolithoautotrophic growth. The growth of the strains was monitored by measuring the absorbance at 600 nm and by determining the concentrations of thiosulfate and sulfate. The cells were harvested by centrifugation and the proteins were extracted. The protein extracts were reduced with dithiothreitol, alkylated with iodoacetamide, and then digested with trypsin. The resulting peptide samples were analyzed by nanoscale liquid chromatography coupled to tandem mass spectrometry (nanoLC-MS/MS) using an Easy nLC1000 liquid chromatography system coupled to a Q-Exactive plus Orbitrap mass spectrometer (Thermo Fisher Scientific, IL, United States). The proteins were identified from the obtained mass spectra using Proteome Discoverer 2.0 (Thermo Fisher Scientific) and in-house databases constructed from the genomes of the respective strains. The abundance of proteins was estimated as the exponentially modified protein abundance index (emPAI) (Ishihama et al., 2005). For each strain, two sets of proteomic data were obtained from independent cultures, and the averaged emPAI values of the respective proteins were calculated from the two datasets. The detected proteins were sorted by the averaged emPAI value and were grouped into four categories based on the following ranking: within the top 2%, 2–10%, 10–30%, and below 30%. More detailed procedures are described in the Supplementary Material.

## Results and Discussion

### Characteristics of Newly Isolated Strains

**FIGURE 2:**
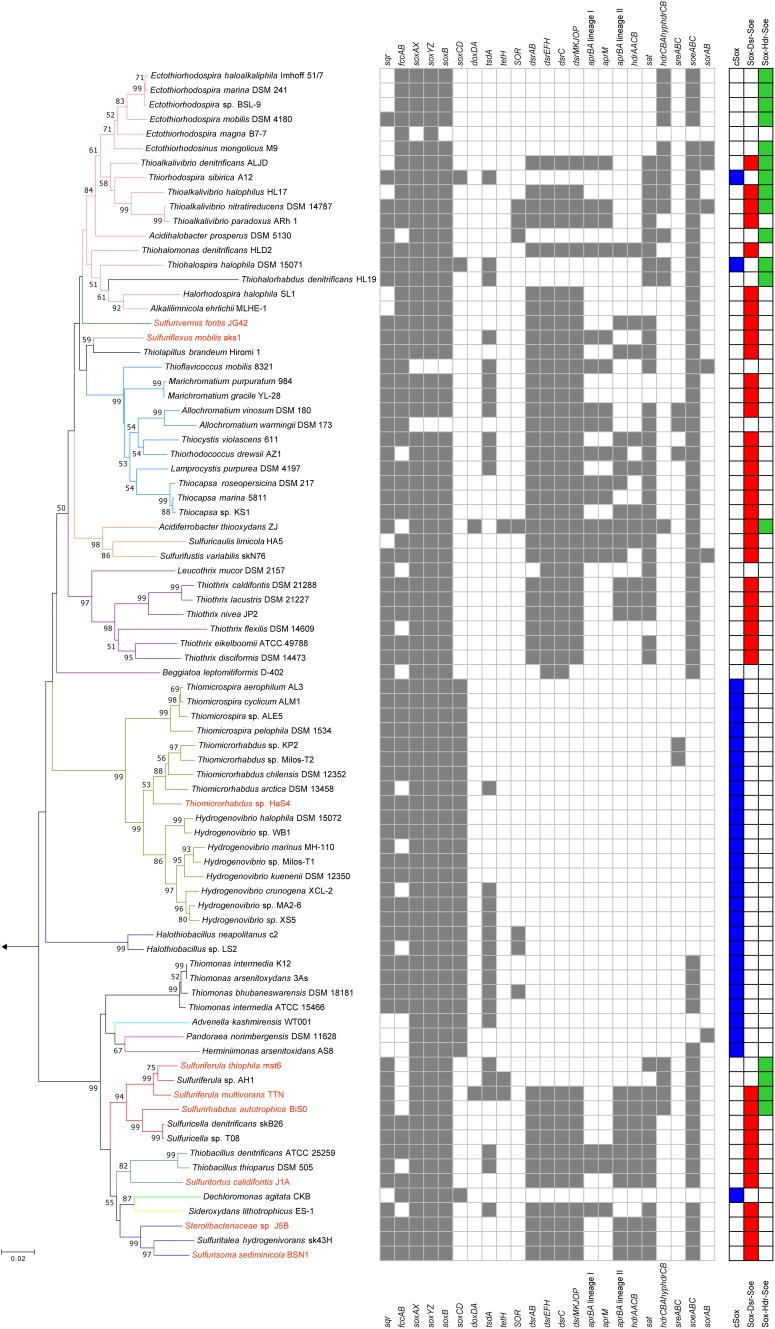
Phylogeny of sulfur-oxidizing bacteria and distribution of genes for sulfur oxidation. Phylogenetic tree was constructed by neighbor-joining method with 16S rRNA gene sequences aligned by ClustalW. The number of final comparable positions was 1224. Bootstrap values (50% >) from 1,000 replicates are shown next to branches. Branches are colorized on the basis of the family-level taxonomy. Strains shown in red are organisms whose genomes were sequenced in this study. The tree was rooted with the 16S rRNA sequence of *Sulfurimonas autotrophica* OK10. Three right-most columns represent distribution of the core sulfur oxidation pathways.

Two novel sulfur oxidizers, designated as strains HaS4 and J5B, were obtained from lake water and a hot spring microbial mat, respectively. The 16S rRNA gene sequence analysis revealed that strain HaS4 belongs to the genus *Thiomicrorhabdus* in the class *Gammaproteobacteria*, but it is distinct from the existing species in this genus, with a sequence similarity lower than 96% (Figure 2). All known *Thiomicrorhabdus* species are obligately chemolithoautotrophic and oxidize inorganic sulfur compounds (Boden et al., 2017b,c). It was also revealed that strain J5B belongs to the family *Sterolibacteriaceae* in the class *Betaproteobacteria* (Figure 2) and potentially represents a novel genus, because none of previously described genera can accommodate this strain (Supplementary Figure S1). The family *Sterolibacteriaceae* was defined on the basis of 16S rRNA gene sequences and currently consists of 6 monospecific genera (Boden et al., 2017a). Among these genera, *Sulfuritalea* and *Sulfurisoma* have been described as facultatively autotrophic sulfur oxidizers which utilize some organic acids.

Strain HaS4 grew at a temperature range of 0–25°C, with optimum growth observed at 22°C. The growth of this bacterium was observed at a pH range of 6.2–8.8, with an optimum range of 6.6–7.4. Strain HaS4 grew chemolithoautotrophically on thiosulfate, tetrathionate, elemental sulfur and sulfide, but not on hydrogen gas. The following organic substrates did not support growth of the strain: lactate, acetate, formate, fumarate, glucose, maltose, fructose, *N*-acetyl-D-glucosamine, sucrose, and cellobiose.

Strain J5B grew at temperature range of 28–55°C, with optimum growth observed at 45–50°C. The growth of this strain was observed at a pH range of 5.8–8.7, with an optimum range of 6.7–7.4. Under nitrate-reducing conditions, strain J5B grew chemolithoautotrophically on thiosulfate and elemental sulfur, but not on sulfide, tetrathionate, or hydrogen gas. Strain J5B grew anaerobically on the following organic substrates in the presence of nitrate: pyruvate, lactate, acetate, propionate, succinate, fumarate, malate, and butyrate. The following substrates could not support anaerobic growth of strain J5B: benzoate, isobutyrate, methanol, ethanol, formate, citrate, glucose, xylose, phenol, *o*-cresol, and *m*-cresol.

### Sequencing and Assembly of Genomes

The basic characteristics of the newly sequenced genomes are summarized in Table 2. The draft genome sequence of *Sulfurisoma sediminicola* BSN1 was obtained with a combination of GS FLX and Sanger sequencing for gap closing. By closing 46 gaps within the scaffolds, 8 contigs were finally obtained. For the *Sulfuriferula* species strains, draft genome sequences were obtained using a HiSeq platform. By assembling paired-end reads, 87 and 48 scaffolds were constructed for the strains TTN and mst6, respectively. Some of these scaffolds are segmented into two or more contigs separated by unclosed gaps, the lengths of which were estimated. Similarly, 178 contigs in 83 scaffolds were assembled for *Sulfurirhabdus autotrophica* BiS0. For the other 5 strains analyzed with PacBio, single circular chromosomes were successfully constructed. Additionally, small circular contigs were obtained for strains J5B and HaS4, suggesting the presence of plasmids in these strains. These results indicated the advantage of using the long-reads produced by PacBio sequencing to obtain complete genome sequences.

### Distribution of Genes for Sulfur Oxidation

The genes for sulfur oxidation identified in the newly sequenced genomes were compared with those of other sulfur oxidizers, and summarized in Figure 2. For the comparative analysis, the genomes of 73 strains isolated in pure culture were obtained as verifiable references, and genes encoding the following proteins were identified in the genomes: Sqr (Griesbeck et al., 2002; Marcia et al., 2009), FccAB (Chen et al., 1994), SoxXZYABCD (Friedrich et al., 2000, 2001), DsrAB (Pott and Dahl, 1998), DsrEFH (Stockdreher et al., 2012), DsrC (Stockdreher et al., 2012), DsrMKJOP (Grein et al., 2010), HdrCBAHypHdrCB (Cao et al., 2018; Koch and Dahl, 2018), SoeABC (Dahl et al., 2013), SorAB (Kappler et al., 2000; Kappler and Bailey, 2005), AprBA (Lyric and Suzuki, 1970; Fritz et al., 2002), AprM (Pires et al., 2003; Meyer and Kuever, 2007; Parey et al., 2013), HdrAACB/QmoABHdrCB (Meyer and Kuever, 2007; Ramos et al., 2012; Watanabe et al., 2014), TsdA (Denkmann et al., 2012; Brito et al., 2015), SOR (Kletzin, 1989, 1992), TetH (De Jong et al., 1997), DoxDA (Müller et al., 2004), and SreABC (Laska et al., 2003). Except for the SreABC, functions of these proteins in sulfur oxidation have been examined in the previous studies listed above. The involvement of SreABC in the reverse reaction of persulfide reductase was suggested in a green sulfur bacterium but has not yet been examined (Eddie and Hanson, 2013).

### Core Sulfur Oxidation Pathways

Although combinations of sulfur oxidation genes are highly diverse among the genomes, typical combinations were identified as a genetic basis for three types of core sulfur oxidation pathways that consisted of the following different sets of enzymes: (1) the “cSox” pathway, with SoxXYZABCD; (2) the “Sox-Dsr-Soe” pathway, with SoxXYZAB, DsrABEFHCMKJOP, and SoeABC; and (3) the “Sox-Hdr-Soe” pathway, with SoxXYZAB, HdrCBAHypHdrCB, and SoeABC (Figure 1, 2). All these core pathways contain SoxXYZAB, and their structural genes were the most commonly observed in the sulfur-oxidizing bacteria analyzed. Wide distribution of *soxXYZAB* has also been shown in a previous study (Meyer et al., 2007). SoxAX catalyzes oxidative conjugation of thiosulfate to cysteine residue on SoxY of the SoxYZ complex, and the sulfonate group is removed by hydrolysis via SoxB with the generation of sulfate and SoxYZ with the sulfane sulfur (Friedrich et al., 2000, 2001; Bamford et al., 2002; Sauvé et al., 2007, 2009). It was also reported that a mixture of the purified SoxAX, SoxYZ, and SoxB enzymes catalyzes sulfite oxidation (Friedrich et al., 2000). Such functional versatility of Sox complex may be related to its wide distribution.

One of the strains isolated in this study, strain HaS4, possesses the cSox pathway consisting of SoxXYZABCD (Figure 2). This pathway is completely conserved in sulfur oxidizers of the family *Piscirickettsiaceae*. Genomic features of these sulfur oxidizers were closely inspected in a recent study, which revealed prevalence of the SoxCD among them (Scott et al., 2018). It also pointed out that genes encoding SoeABC are absent from their genomes. The comparative analysis in the present study revealed that sulfur oxidizers with the cSox pathway generally lack SoeABC (Figure 2), one of the most common sulfite-oxidizing enzyme complexes in the analyzed strains. The SoxCD complex oxidizes the sulfane sulfur of SoxZY-Cys-S^-^ derived from the SoxXYZAB reaction using water molecules to yield the sulfonate group as SoxZY-Cys-SO_3_^-^ (Quentmeier et al., 2000; Zander et al., 2010), which is further hydrolyzed to free sulfate ion by SoxB. In a revised model of Sox reaction recently proposed, sulfur carrier for the SoxCD reaction is SoxZY-Cys-S(n)-S^-^ rather than SoxZY-Cys-S^-^ (Grabarczyk and Berks, 2017). The cytoplasmic sulfite oxidase SoeABC may not be important for organisms with SoxCD because SoxXYZABCD completely oxidizes thiosulfate to sulfate in the periplasm (cSox means “complete Sox system”). Interestingly, it turned out that *Thiorhodospira sibirica* A12 and *Thiohalospira halophila* DSM 15071 have *soeABC* in addition to *soxCD* (Figure 2). These sulfur oxidizers also possess the *hdrCBAhyphdrCB* gene cluster, which is always accompanied by *soeABC*, as discussed below.

The Sox-Dsr-Soe pathway is the most common core sulfur oxidation pathway in the newly sequenced genomes from this study (Figure 2). In this pathway, sulfane sulfur derived from thiosulfate via SoxXYZAB is transported to the cytoplasm presumably in the form of persulfides, as suggested by a phototrophic sulfur oxidizer (Frigaard and Dahl, 2008). Persulfide sulfur is then transferred to DsrC via Rhd, TusA, and DsrEFH (Cort et al., 2008; Stockdreher et al., 2012, 2014). The persulfurated form of DsrC is considered to be the substrate for DsrAB, as shown by the crystal structure of the DsrABC complex from *Desulfovibrio vulgaris* (Oliveira et al., 2008). The siroheme-containing cytoplasmic enzyme DsrAB is involved in the reverse reaction of reduction of sulfite to sulfide as demonstrated in sulfate-reducing prokaryotes (Schedel et al., 1979; Pott and Dahl, 1998); generates sulfite and disulfide bond in DsrC, which is reduced to free thiols by the DsrMKJOP transmembrane complex for restart of the sulfur relay system (Dahl et al., 2005; Pires et al., 2006; Grein et al., 2010). The resulting sulfite is oxidized to sulfate by the cytoplasmic enzyme complex SoeABC (Dahl et al., 2013). In this study, it was found that the genes encoding the SoeABC complex are completely conserved in the *dsr*-positive sulfur oxidizers (Figure 2). The reactions catalyzed by Dsr and SoeABC both occur in the cytoplasm (Figure 1), and this colocalization may be one of the reasons for the coexistence of the genes encoding Dsr and SoeABC in the genomes. In theory, sulfite generated by DsrAB can be oxidized by Sox proteins (Friedrich et al., 2000), but transport of the sulfite across the cytoplasmic membrane would be required in this case since the Sox reaction occurs in the periplasm (Figure 1).

The other core sulfur oxidation pathway is Sox-Hdr-Soe. This pathway essentially consists of the proteins of the Sox-Dsr-Soe pathway, but Dsr proteins are replaced with Hdr proteins that are encoded in the gene cluster *hdrCBAhyphdrCB* (Figure 1). Recently, the role of these Hdr proteins in sulfur oxidation was genetically investigated with *Hyphomicrobium denitrificans*, a bacterium which degrades dimethylsulfide (DMS) (Koch and Dahl, 2018). The mutagenesis of *hdr* genes resulted in loss of ability to metabolize DMS and a lower rate of the sulfate formation from thiosulfate than wild type under chemoorganoheterotrophic growth conditions. Although the reaction catalyzed by HdrCBAHypHdrCB has not yet been revealed, involvement of these proteins in the sulfite generation from persulfides was proposed based on sequence similarities of *hdr* genes with archaeal counterparts along with mutagenesis experiments. In thiosulfate metabolism, HdrCBAHypHdrCB might functionally substitute for Dsr system as shown in Figure 1. As is the case with the *dsr* genes, all the analyzed genomes with *hdrCBAhyphdrCB* also harbor *soeABC* genes.

The majority of the strains analyzed have one of the three core pathways for sulfur oxidation, although some exceptions were noted, as described below. Among the 82 strains included in the comparative analysis (shown in Figure 2), three strains lack some or all of the genes encoding SoxXYZAB, the common component of the three core pathways. The strains *Ectothiorhodospira magna* B7-7, *Thioflavicoccus mobilis* 8321, and *Allochromatium warmingii* DSM173 are sulfur oxidizers that utilize sulfur compounds other than thiosulfate. *Leucothrix mucor* DSM2157 and *Beggiatoa leptomitiformis* D-402 harbor the genes encoding SoxXYZAB and SoeABC but lack those encoding conserved proteins in the core pathways. In contrast to these 5 strains lacking components for the core pathways, some other strains were observed to have full gene sets for two core pathways. It has been noted that the *dsr* and *hdrCBAhyphdrCB* genes are almost exclusive to each other, but a small number of organisms have both genes in their genomes (Koch and Dahl, 2018). This notable overlap was observed in the genomes of *Sulfurirhabdus autotrophica* BiS0 and *Sulfuriferula multivorans* TTN, which were sequenced in this study. In addition, a recent study revealed that three *Acidiferrobacter* strains have *dsr* and *hdrCBAhyphdrCB* along with *soeABC* (Issotta et al., 2018). These findings indicate that these strains have two core pathways, Sox-Dsr-Soe and Sox-Hdr-Soe. As mentioned above, *Thiorhodospira sibirica* A12 and *Thiohalospira halophila* DSM 15071 harbor both *soxCD* and *hdrCBAhyphdrCB*, representing organisms with cSox and Sox-Hdr-Soe pathways. In this study, no organism possessing both the cSox and Sox-Dsr-Soe pathways was identified. The *soxCD* and *dsr* genes are well known to be mutually exclusive (Meyer and Kuever, 2007; Frigaard and Dahl, 2008; Gregersen et al., 2011; Lenk et al., 2012).

### Variations in Sulfur Oxidation Pathways Within the Family ‘*Sulfuricellaceae*’

**FIGURE 3:**
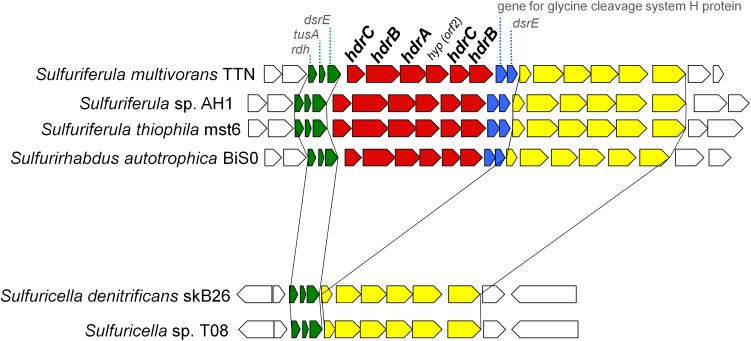
Gene arrangement around *hdrCBAhyphdrCB* gene cluster in the genomes of ‘*Sulfuricellaceae*’. Genes of *hdrCBAhyphdrCB* cluster are shown in red. Genes shown in blue are conserved along with the *hdr* genes in the ‘*Sulfuricellaceae*’. Genes shown in green and yellow are conserved in the all ‘*Sulfuricellaceae*’ genomes in the same order and direction.

The patterns for the occurrence of the core sulfur oxidation pathways were generally consistent with the 16S rRNA gene-based phylogeny (Figure 2). In other words, phylogenetically close organisms share a similar genetic basis for sulfur oxidation. However, there were some notable deviations from this tendency. In particular, considerable variations were observed among the genomes of ‘*Sulfuricellaceae*’ strains. In this family, 6 genomes of isolated strains were available for the analysis, including three genomes obtained in this study. *Sulfuriferula thiophila* mst6 and *Sulfuriferula* sp. AH1 lack the *dsr* operon, which is present in all the other members of the family. In contrast, two *Sulfuricella* strains lack the *hdrCBAhyphdrCB* cluster, which all the other strains possess. Consequently, 6 ‘*Sulfuricellaceae*’ strains were classified into three types, two strains with the Sox-Dsr-Soe pathway, two strains with the Sox-Hdr-Soe pathway, and two strains with both pathways. A close inspection of the genomes of these bacteria revealed that the *hdrCBAhyphdrCB* cluster is located between genomic regions that are conserved among these strains, and these conserved regions are directly adjacent to each other in the genomes of *Sulfuricella* strains lacking this gene cluster (Figure 3). This observation may indicate that a common ancestor of ‘*Sulfuricellaceae*’ had *hdrCBAhyphdrCB* and that *Sulfuricella* strains lost this cluster during evolution. The coexistence of the *hdrCBAhyphdrCB* and *dsr* genes in *Sulfuriferula multivorans* TTN and *Sulfurirhabdus autotrophica* BiS0 may represent a transition state, and one of them may be selected from the genomes in the future.

Another example of differences observed among organisms in this family is that *Sulfuriferula multivorans* TTN has *doxDA* gene encoding a membrane-bound thiosulfate:quinone oxidoreductase, which couples oxidation of thiosulfate to reduction of quinone (Müller et al., 2004). DoxDA is one of the most minor proteins involved in sulfur oxidation in the reference strains (Figure 2). The gene encoding DoxDA was only identified in the acidophilic gammaproteobacteria *Acidihalobacter ferrooxidans* and *Acidiferrobacter thiooxidans* (Valdés et al., 2008; Quatrini et al., 2009; Issotta et al., 2018).

### Expression of Proteins Involved in Sulfur Oxidation

**FIGURE 4:**
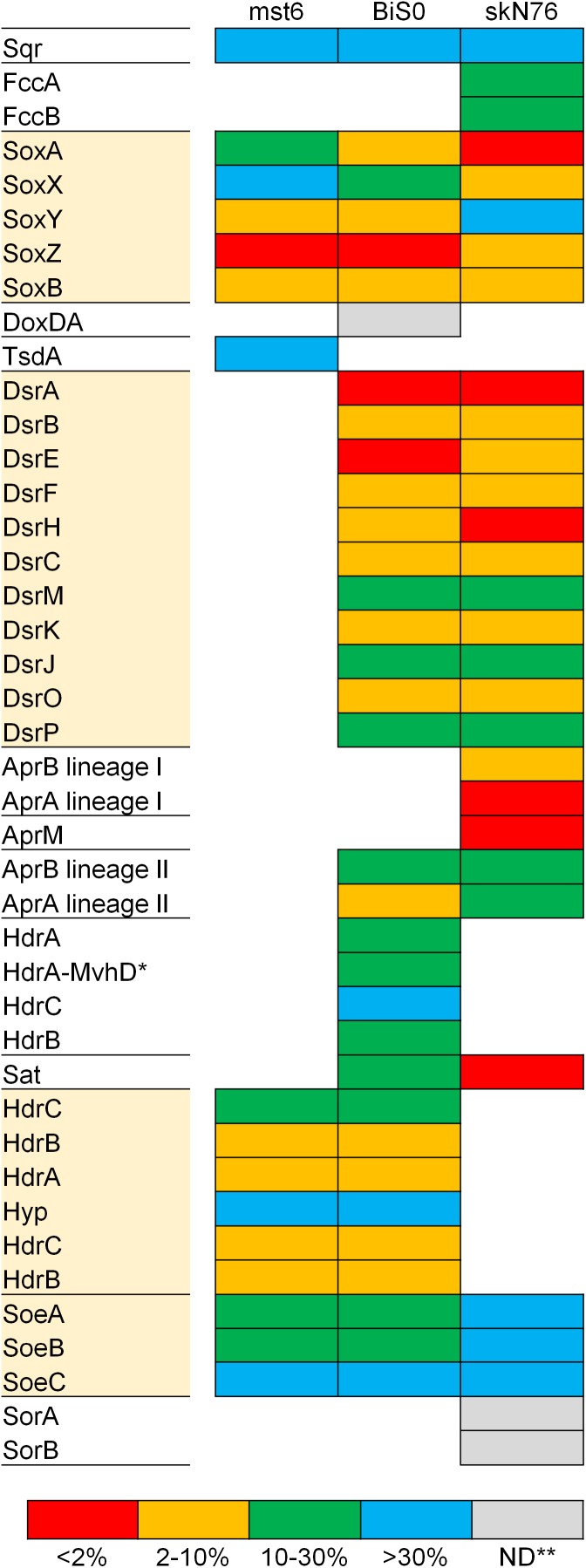
Expression levels of proteins for sulfur oxidation estimated with proteomic analysis of *Sulfuriferula thiophila* mst6, *Sulfurirhabdus autotrophica* BiS0 and *Sulfurifustis variabilis* skN76 grown on thiosulfate. The detected proteins were grouped into four categories based on ranking of abundance (see text for detail). Proteins highlighted with shaded boxes are components of the core sulfur oxidation pathway. ^∗^HdrA-MvhD, HdrA fused with MvhD; ^∗∗^ND, not detected.

The expression of the proteins required for sulfur oxidation was investigated for the whole proteomes of three sulfur oxidizers with different sets of the core pathways, including *Sulfuriferula thiophila* mst6 with Sox-Hdr-Soe, *Sulfurifustis variabilis* skN76 with Sox-Dsr-Soe, and *Sulfurirhabdus autotrophica* BiS0 with both. In the proteomes of the strains grown on thiosulfate, proteins corresponding 41–48% of the total protein-coding genes predicted in the corresponding genomes were detected. In all the strains, the summed abundance of the top 10% most abundant proteins represented more than 90% of the total protein abundance (Supplementary Figure S2). As shown in Figure 4, almost all the proteins involved in sulfur oxidation were detected in the proteomes. Majority of the Sox, Dsr, and Hdr proteins were among the top 10% most abundant proteins. In addition to these major components of the core pathways, some additional proteins were also detected among the most abundant proteins in strain skN76.

Compared to the Sox and Dsr proteins, evidence for the essential contribution of the Hdr proteins encoded by *hdrCBAhyphdrCB* in sulfur oxidation had been limited. The involvement of this gene cluster in sulfur oxidation was previously investigated by transcriptomic approaches using *Acidithiobacillus* strains (Quatrini et al., 2009; Chen et al., 2012). In the transcriptomes of these strains fed elemental sulfur or tetrathionate, the transcripts from this gene cluster were detected except for that of *orf2* (referred to as *hyp* in this study) located between *hdrCBA* and *hdrCB*. A recent study proposed the involvement of HdrCBAHypHdrCB in generation of sulfate from thiosulfate via the sulfite formation from persulfides (Koch and Dahl, 2018). In the present study, the products of this gene cluster were detected in *Sulfuriferula thiophila* mst6 and *Sulfurirhabdus autotrophica* BiS0 grown on thiosulfate. These results support the involvement of these proteins in thiosulfate-dependent chemolithoautotrophic growth.

*Sulfurirhabdus autotrophica* BiS0 has both the Sox-Dsr-Soe and Sox-Hdr-Soe pathways. A previous study of *Hyphomicrobium* suggested that HdrCBAHypHdrCB catalyzes the oxidation of protein-bound persulfide sulfur to generate sulfite and that this reaction can functionally substitute for that catalyzed by the Dsr proteins (Koch and Dahl, 2018). In the *Sulfurirhabdus autotrophica* BiS0 proteome, the products of both the *dsr* and *hdrCBAhyphdrCB* operons were detected at a relatively high abundance (Figure 4). These results suggest that strain BiS0 uses both systems to generate sulfite. The biological importance of the apparent simultaneous expression of these proteins is currently unclear. This subject may be an important key to understanding the evolution of sulfur-oxidizing systems, because many sulfur oxidizers have two or more genes performing the same physiological function. Such functional overlap was prominently observed in the genome and proteome of *Sulfurifustis variabilis* skN76. This strain has two copies each of *dsrAB*, *aprBA* and *fccAB* genes (Umezawa et al., 2016). In its proteome, encoded products of these genes were all detected (Figure 4).

### Genes for Arsenite Oxidation and Respiratory Arsenate Reduction

Some prokaryotes, including sulfur oxidizers, are known to have genes encoding either of the two types of arsenite oxidases, Aio or Arx (van Lis et al., 2013). The *arxAB* genes, which encode catalytic units of the Arx, have been identified in a limited number of bacteria that primarily belong to the class *Gammaproteobacteria* (Zargar et al., 2012; Ospino et al., 2018). In this study, the genomes of *Sulfuritortus calidifontis* J1A and strain J5B were observed to harbor the *arxAB* genes. Among sulfur oxidizers, the *arxAB* genes have been only identified in members of the family *Ectothiorhodospiraceae* isolated from salty alkaline environments. As an exception, the genes were identified in a plasmid from *Sulfuricella denitrificans* skB26, which is a neutrophilic betaproteobacterium belonging to the family ‘*Sulfuricellaceae*’ (Watanabe et al., 2014). The strains J1A and J5B are neutrophilic betaproteobacteria isolated from a same microbial mat, but they belong to the families *Thiobacillaceae* and *Sterolibacteriaceae*, respectively. In the present study, the genomes of four other strains belonging to the families ‘*Sulfuricellaceae*’ and *Sterolibacteriaceae* were also sequenced (Table 1) but the *arxAB* genes were identified in none of them. In contrast to strain skB26, the *arxAB* genes of strains J1A and J5B are located in their chromosomes. Interestingly, strain J1A also has the *aioAB* genes encoding catalytic units of Aio in its genome. To the best of our knowledge, this is the first report describing the coexistence of *aioAB* and *arxAB* genes in single bacterial strain isolated in pure culture. It will be subject of future work to identify roles of these two oxidases in arsenic metabolism of the strain J1A.

*Sulfuritalea hydrogenivorans* sk43H is the first betaproteobacterium for which the ability for arsenate respiration was demonstrated, and it has *arrAB* genes encoding respiratory arsenate reductase (Watanabe et al., 2017). In this study, the genomes of its relatives within the same family were sequenced. Although *Sulfurisoma sediminicola* BSN1 and strain J5B have physiological traits that are similar to those of strain sk43H (e.g., facultatively anaerobic, facultatively autotrophic, neutrophilic), the *arrAB* genes were not observed in their genomes. These observations of the *arx* and *arr* indicate that the presence of these genes in genomes cannot be predicted by phylogenetic proximity.

## Conclusion

In this study, the genomes of 9 sulfur-oxidizing bacteria were sequenced. These sulfur oxidizers belong to 8 genera, including 6 for which no genome sequence of a cultured organism was available. In the comparative genome analysis, typical suites of genes were identified for core sulfur oxidation pathways. The results of the analysis suggested the crucial importance of the cytoplasmic sulfite oxidase encoded by *soeABC* in the sulfur oxidizers without *soxCD*. In addition, large variations in the sulfur oxidation pathways were observed among members of the family ‘*Sulfuricellaceae*’. Furthermore, the *arx* genes were discovered in the families *Thiobacillaceae* and *Sterolibacteriaceae*. These findings provide some insights into the mechanism and evolution of sulfur metabolism and expand knowledge of arsenite oxidases. The primary significance of this study may lie in providing the genome sequences of 9 sulfur oxidizers, which are certainly linked to the identity and physiology of the respective organisms. These genomes will serve as valuable references for various kinds of sequence-based analyses including amplicon sequencing of various genes, metagenomics, metatranscriptomics and metaproteomics.

## Author Contributions

TW, HK, and MF designed the study. HK and YK isolated and characterized strains. TW and HK performed experiments for genome sequencing. TW, KU, CH, and TT carried out proteomic experiments. TW conducted data analyses. TW and HK wrote the manuscript.

## Conflict of Interest Statement

The authors declare that the research was conducted in the absence of any commercial or financial relationships that could be construed as a potential conflict of interest.

## Footnotes

1 http://gtdb.ecogenomic.org/
