# Supplementary material for: Genomes of Neutrophilic Sulfur-Oxidizing Chemolithoautotrophs Representing 9 Proteobacterial Species From 8 Genera
Source: Front Microbiol. 2019 Feb 25;10:316. doi: 10.3389/fmicb.2019.00316 (PMC6397845; doi:10.3389/fmicb.2019.00316)
Supplement: Supplementary file 1 [file Data_Sheet_1.PDF]

Supplementary Information

Genomes of neutrophilic sulfur-oxidizing chemolithoautotrophs representing 9 proteobacterial species from 8 genera

Tomohiro Watanabe, Hisaya Kojima*, Kazuhiro Umezawa, Chiaki Hori, Taichi E. Takasuka, Yukako Kato and Manabu Fukui

***Correspondence:** Hisaya Kojima: kojimah@pop.lowtem.hokudai.ac.jp

Detailed experimental procedures for physiological characterizations and proteomic analysis

Isolation and characterization of novel sulfur-oxidizing strains

Strain HaS4 was isolated from water of a meromictic lake in Japan, Lake Harutori (Kubo et al., 2016). Water column of Lake Harutori consists of two layers with different salinity, and the sample was obtained from bottom of low-density surface water layer. During the process of enrichment and isolation of strain HaS4, medium S4 (Kojima et al., 2016) supplemented with 20 mM $Na_2S_2O_3$ was used. The first enrichment culture was established at 22°C by inoculating the lake water into the medium. The strain was isolated in pure culture by repeated serial dilution in the same medium. The isolated strain was cultured at various temperatures (0, 5, 8, 15, 18, 22, 25, 28, 30, 32 and 37°C) to test temperature dependency of the growth. The effect of pH on the growth was tested as described previously (Kojima *et al*., 2015) with a slightly modified medium which contained no vitamins. The tested pH and buffering reagents were as follows; pH 6.1, 6.2, 6.3, 6.6 and 6.8 with MES; pH 6.8, 7.0 and 7.1 with PIPES; pH 7.3, 7.4, and 7.7 with MOPS; pH 8.0 and 8.2 with Tricine; pH 8.8, 9.1, 9.3

and 9.6 with CHES. To investigate utilization of growth substrate, a modified version of medium S4 was prepared by excluding $Na_2S_2O_3$ and increasing $MgSO_4.7H_2O$ concentration to 0.5 g l^{-1}. The modified medium was supplemented with one of the following substrates as sole electron donor: thiosulfate (10–20 mM), tetrathionate (10 mM), elemental sulfur (0.5 g l^{-1}), sulfide (2 mM), hydrogen gas (air/H_2 80:20 v/v; 125 kPa in total pressure), lactate (10 mM), acetate (10 mM), formate (10 mM), fumarate (5 mM), glucose (5 mM), maltose (5 mM), fructose (5 mM), *N*-acetyl-D-glucosamine (2 mM), sucrose (2 mM), and cellobiose (1 mM). Anaerobic growth of the strain was tested with the medium S4 amended with $Na_2S_2O_3$ and $NaNO_3$ (10 mM each) under anoxic condition created by filling the headspace of the culturing bottles with mixed gas (N_2/CO_2; 80:20 v/v).

Strain J5B was isolated from an enrichment culture grown on elemental sulfur, from which *Sulfuritorta calidifontis* J1A and *Sulfurivermis fontis* JG42 were isolated (Kojima et al., 2017a, 2017b). The enrichment culture was established from a microbial mat obtained from Jozankei hot spring in Japan. From the enrichment culture, a small portion was transferred to the medium S5 (Kojima et al., 2017b) supplemented with 10 mM $NaNO_3$ and cultured at 50°C under anoxic condition. From the resulting thiosulfate-oxidizing nitrate-reducing culture, strain J5B was obtained with repeated agar shake dilution using the same medium under anoxic conditions at 45°C. Strain J5B was cultured at various temperatures (5, 8, 10, 13, 15, 18, 22, 25, 28, 30, 32, 35, 37, 42, 45, 48, 50, 55, 60 and 60°C) with the medium supplemented with 5 mM sodium acetate and 10 mM $NaNO_3$, in closed bottles with headspace filled with N_2/CO_2 (80:20; v/v). Effects of pH on growth of strain J5B were examined under nitrate-reducing conditions, with a method modified from that previously described (Kojima et al., 2015). Briefly, the composition of the basal medium was changed as follows (l^{-1}): 2 g $Na_2S_2O_3.5H_2O$, 1 g $NaNO_3$, 0.3 g $MgSO_4$. $7H_2O$, 0.1 g $CaCl_2$. $2H_2O$, 0.1 g NH_4Cl, 0.1 g KH_2PO_4, 0.1 g KCl, 1 g disodium succinate, and 0.1 g yeast extract. It also contained solutions of trace elements, selenite-tungstate, and vitamin mixture as same in the original medium. Depending

on the final pH, one of the buffering reagents was added to the basal medium to obtain final concentration of 20 mM, as follows; pH 4.8, 5.0, 5.3, 5.4, 5.8, 6.0 and 6.4 with MES; pH 6.4, 6.7 and 7.2 with PIPES; pH 6.9, 7.1, and 7.3 with MOPS; pH 7.4, 7.9, 8.1, 8.3, 8.4 and 8.7 with Tricine; pH 8.3, 8.7, 9.1, 9.2, 9.4, 9.5 and 9.7 with CHES. All ingredients were mixed and then sterilized by filtration, after pH adjustment with HCl (media of pH 4.8–5.3) or NaOH (all the other media). The sterilized media were dispensed in closed culture bottles whose headspace was filled with N_2 gas. The bottles inoculated with the strain were incubated as 45°C. Aerobic growth under the air was tested with S5 supplemented with 10 mM S_2O_3 at 45°C. Utilization of electron donor was tested under anoxic conditions as 45°C, by using modified S5 medium which contained 10 mM $NaNO_3$ and no thiosulfate. The medium contained 5 g $Na_2S_2O_3.5H_2O$ and 0.2 g $MgCl_2.6H_2O$ instead of Mg $SO_4.7H_2O$ in medium S5. The tested electron donors were as follows: thiosulfate (10 mM), (0.5 g l^{-1}), sulfide (2 mM), tetrathionate (10 mM), hydrogen gas ($H_2/N_2/CO_2$ 50:40:10 v/v/v; 200 kPa in total pressure), pyruvate (5 mM), lactate (5 mM), acetate (5 mM), propionate (2.5 mM), succinate (2.5 mM), fumarate (2.5 mM), malate (2.5 mM), butyrate (2.5 mM), benzoate (2.5 mM), isobutyrate (2.5 mM), methanol (5 mM), ethanol (2.5 mM), formate (5 mM), citrate (5 mM), glucose (2.5 mM), xylose (2.5 mM), phenol (2 mM), *o*-cresol (1 mM), *m*-cresol (1 mM).

Proteomic analysis

Sulfuriferula thiophila mst6, *Sulfurirhabdus autotrophica* BiS0 and *Sulfurifustis variabilis* sKN76 were cultured under oxic conditions, at 30˚C, 22˚C and 45˚C respectively. The strains were cultured in a bicarbonate-buffered medium which contains thiosulfate as sole electron donor for chemolithoautotrophic growth. The composition of the medium was as follows (l^{-1}): 10 g $Na_2S_2O_3$. $5H_2O$, 0.2 g $MgCl_2$. $6H_2O$, 0.1 g $CaCl_2$. $2H_2O$, 0.1 g NH_4Cl, 0.1 g KH_2PO_4, 0.1 g KCl, 1 ml trace element solution, 1 ml selenite-tungstate solution, 1 ml vitamin mixture solution and 30 ml $NaHCO_3$ solution. The vitamin mixture contained following constituents (l^{-1}); 2 mg biotin, 2 mg folic acid, 10 mg pyridoxine-HCl, 5 mg thiamine-HCl. $2H_2O$, 5 mg riboflavin, 5 mg nicotinic acid, 5 mg calcium

D(+) pantothenate, 5 mg 4-aminobenzoic acid, 5 mg lipoic acid, and 0.1 mg cyanocobalamine. All other stock solutions were prepared as described previously (Widdel & Bak, 1992). The final pH was adjusted with HCl to 7.0–7.1. Sulfur-oxidizing activity of the strains were monitored by measuring concentrations of thiosulfate and sulfate, quantified by using an ion chromatograph (DX-120; Dionex, Sunnyvale, CA) equipped with a column for anion analyses (IonPac AS4ASC; Dionex). For each strain, two independent cultures were prepared as duplicates.

Grown cells were harvested by centrifugation and washed with phosphate-buffered saline. The washed cells were subjected to protein extraction using ReadyPrep protein extraction kit (total protein) (Bio-Rad). Sonication was performed on ice 10 times for 10 s with 30-s intervals, and lysates were centrifuged by 16,000 x g for 30 min at 20˚C. The proteins in the supernatant were collected by acetone precipitation and then redissolved in lysis solution (8 M urea in 50 mM NH_4HCO_3). The protein concentration in the solution was quantified using Bio-Rad protein assay kit (Bio-Rad Laboratories), and 20 μg of protein was subjected to trichloroacetic acid precipitation. The resulting precipitate was washed with ice-cold acetone for three times, and then resuspended in 1 M urea in 25 mM NH_4HCO_3 buffer. Protein samples were then reduced for 30 min at 50℃ in 5 mM DTT, followed by alkylation with 15 mM iodoacetamide for 30 min at room temperature in the dark. Samples were then digested by adding proteomics grade trypsin (Roche, Germany) at a 1:100 trypsin/protein ratio for 10 hrs at 37℃. Desalination and purification of the resulting peptide samples were performed using a C18 Zip Tip (Merck), with 0.1 % formic acid as elution solution. Mass spectrum was obtained by using Easy nLC1000 hooked up with Q-Exactive plus Orbitrap mass spectrometer (Thermo Fisher Scientific, IL, USA), operated with Xcalibur software (ver. 3.1, Thermo Fisher Scientific). The peptides were separated on a C18 capillary tip column (NTCC-360/75-3-125, Nikkyo Techno, Japan) by linear gradient over 120 min, from 5 to 30 % acetonitrile in 0.1 % formic acid. Full scan mass spectra were obtained with a scan range of 300.0 to 2,000.0 m/z and a resolution of 70,000. Proteins were identified by searching the mass spectra

against CDSs of the genomes, using Proteome Discoverer 2.0 (Thermo Fisher Scientific). The peptide mass tolerance and fragment mass tolerance were set at 10 ppm and 0.8 Da, respectively. The peptide charge was set at +2, +3 and +4. The accuracy and sensitivity of peptide identification were attempted by automatic decoy function and percolator function built in the Proteome Discoverer software. For each strain, proteins detected in both replicates were regarded to be expressed proteins and subjected to the following procedures.

Abundance of each expressed protein was evaluated based on the exponentially modified protein abundance index (emPAI), automatically generated by the software. The emPAI values were once converted to protein abundance index (PAI), according to equation previously described (Ishihama et al., 2005). For each protein, PAI values from two independent cultures were averaged, and obtained value was converted back into the form of emPAI using the same equation. The resulting value of averaged emPAI was used as an index to represent abundance of each protein. For each strain, all expressed proteins were sorted by the averaged emPAI and grouped into four categories based on the ranking: within top 2%, 2-10%, 10-30%, below 30%.

Table S1. List of query sequences used to identify genes for sulfur oxidation.

Gene	Query (locus tag)
sqr	Acife_2028, Acife_2633, Alvin_2145, Acife_2600, Alvin_1195
fccAB	Alvin_1093, Alvin_1092
soxAX	Alvin_2169, Alvin_2168
soxYZ	Alvin_2111, Alvin_2112
soxB	Alvin_2167
soxCD	Tcr_0156, Tcr_0157
doxDA	AFE_0044
tsdA	Alvin_0091, Tint_2892
tetH	AFE_0029
sor	Acife_2601
dsrAB	Alvin_1251, Alvin_1252
dsrEFH	Alvin_1253, Alvin_1254, Alvin_1255
dsrC	Alvin_1256
dsrMKJOP	Alvin_1257, Alvin_1258, Alvin_1260, Alvin_1261, Alvin_1262
aprAB	Alvin_1121, Alvin_1120
aprM	Alvin_1119
hdrAACB	SCL_2523, SCL_2522, SCL_2520, SCL_2521
hdrCBAxhdrCB	CAP_31_RS01855
sat	Alvin_1118
sreABC	Alvin_1317, Alvin_1318, Alvin_1319
soeABC	Alvin_2489, Alvin_2490, Alvin_2491
sorAB	SVA_1391, SVA_1390

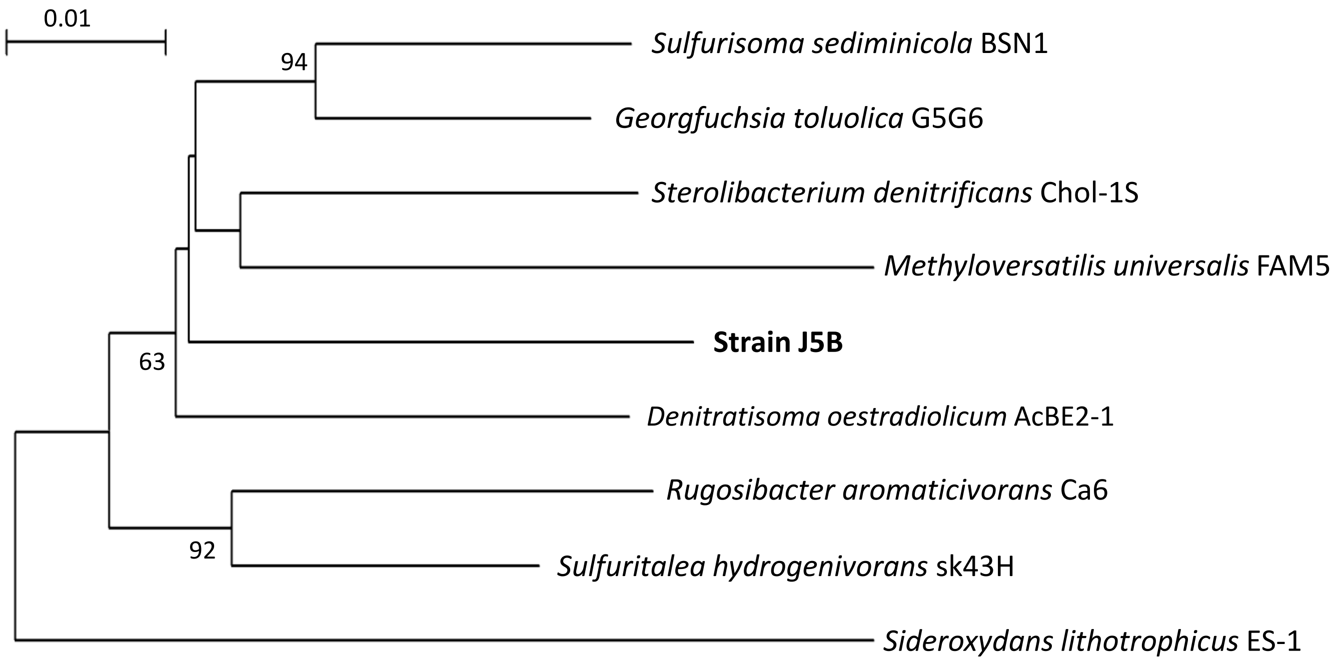

Figure S1. Minimum evolution tree showing the phylogenetic position of strain J5B within the family *Sterolibacteriaceae*. *Sideroxydans lithotrophicus* ES-1 is included as an out group. The genus *Rugosibacter* is currently classified as a member of the family *Rhodocyclaceae.* The other genera belong to *Sterolibacteriaceae*, and all known genera of the family is included in this tree. The tree was constructed by using 1437 sites in the 16S rRNA gene sequences. Numbers at ncurrently classified odes represent percentage values of 500 bootstrap resamplings (values lower than 50 are not shown).

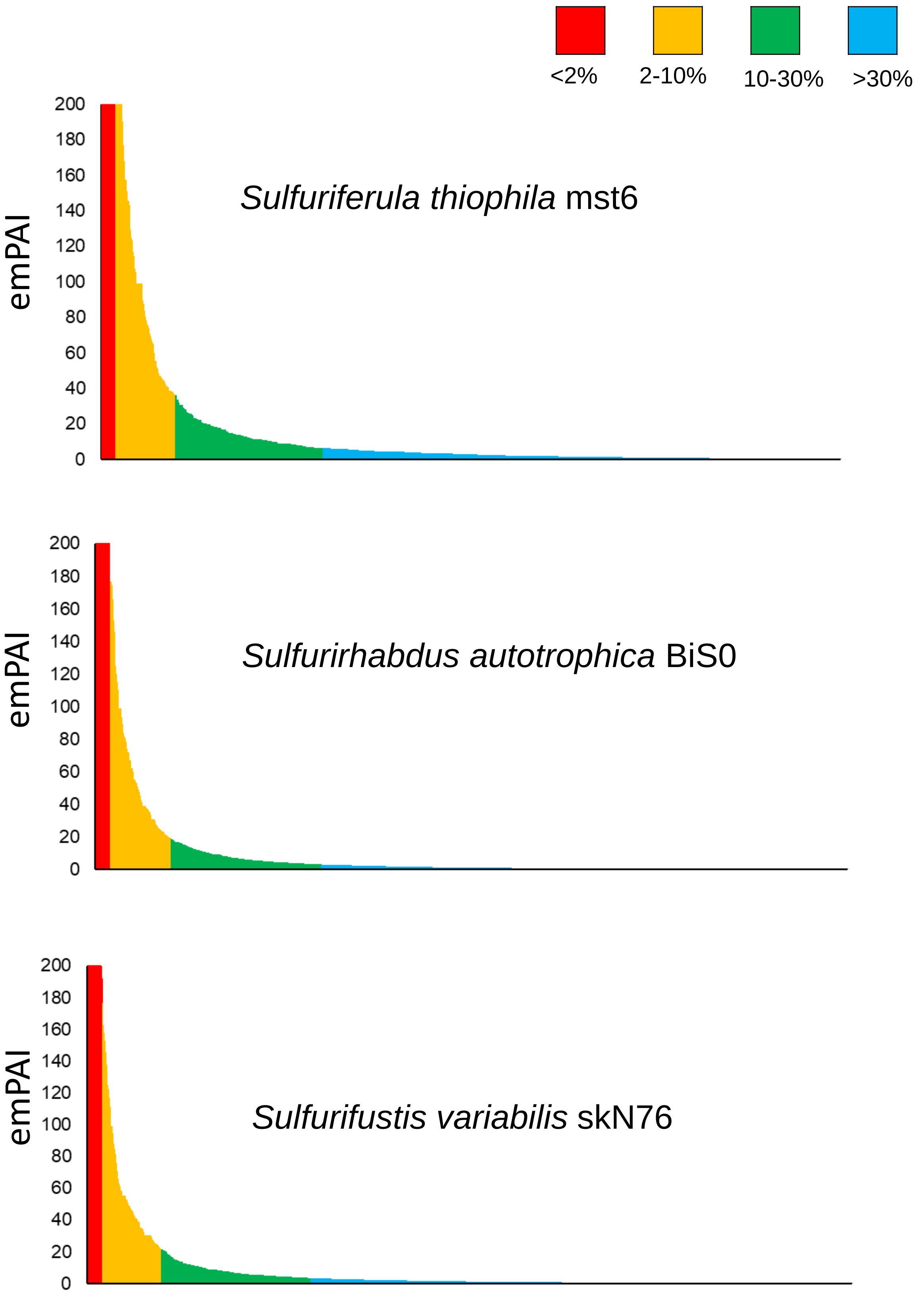

Figure S2. Overviews of the proteomic analysis results. All detected proteins are sorted according to their abundances in each sample expressed as emPAI (values greater than 200 are not shown). Colors indicate four categories based on the ranking and correspond to the colors used in Fig. 4 respectively.
